# Low-Flow Low-Gradient and Low-Ejection Fraction Aortic Stenosis and Projected Aortic Valve Area Calculation: So Important but so Complicated. Let us Just Keep it Simple!

**DOI:** 10.5935/abc.20180030

**Published:** 2018-02

**Authors:** Wilson Mathias Junior

**Affiliations:** Faculdade de Medicina da USP; InCor-HC-FMUSP, São Paulo, SP - Brasil

**Keywords:** Aortic Valve Stenosis / surgery, Stroke Volume, Echocardiogrphy, Stress / methods

Low-flow low-gradient aortic stenosis with low ejection fraction is still one of the main challenges not only for echocardiography but to cardiology itself. It is the very late stage of aortic stenosis that portends very poor prognosis with medical treatment, in addition to a very high operative mortality.^1^ In subjects with that condition, dobutamine stress echocardiography is of paramount importance to stratify aortic stenosis status (real aortic stenosis vs pseudo aortic stenosis) and to predict surgical mortality by the evaluation of the left ventricular contractile reserve status.^1-3^

To better differentiate both parameters, the sole use of the variation of the absolute values of aortic valve area and flow through the outflow tract carries major problems due to load conditions, previous use of medication, such as betablockers, and submaximal stress. All of these limitations may impede the detection of maximal cardiac output, a marker of contractile reserve and, therefore, may underestimate the aortic valve area.

In this regard, the use of the projected aortic valve area tends to correct these limitations and helps us to better predict the patients who tend to get the best benefit from surgery and those who would be less harmed using medical management. Unfortunately, the current formula proposed initially by Blais et al. is cumbersome and of difficult use in clinical practice, especially in high volume centers.^4^ Despite the fact that the current equation was already simplified,^5^ the calculation of flow, in addition to burdensome, may induce to additional errors, because it involves many parameters, such as left ventricular outflow tract (LVOT) diameter and ejection time, and LVOT velocity time integral.

In this regard we welcome the work by Ferreira et al.^6^ in this issue of *Arquivos Brasileiros de Cardiologia*. By using the simplified flow rate calculation (bellow), they could reach a very high concordance with the classical approach. They found that, on average, the alternative method overestimated the projected aortic valve area in 0.037 cm^2^ when comparing to the classic method (95% CI: 0.004-0.066), a variation that is clearly not clinically significant, because this error is lower than 0.1 cm^2^. Their work is not final though, because their findings are mainly based on the analysis of nine patients.

Therefore, when studying a patient with low-flow, low-gradient and low-ejection fraction aortic stenosis, one should always keep in mind the formulas and the explanatory diagram below, to better stratify this very difficult group of patients.^7^ Here is a situation where a carefully performed study may make a difference between life and death. It should be performed by all in all studies! So, let us just keep it simple!

**Alternative flow calculation formula:**

**Q_alternative = AST _LVOT_ × (Vmean _LVOT_ × 100)**

where Q is flow in mL/s, AST_LVOT_ is the sectional transverse area of the left ventricular outflow tract (LVOT) in cm^2^, and Vmean_LVOT_ is the mean blood flow velocity by pulsed wave Doppler at the LVOT level during left ventricular ejection, being expressed in m/s.

**Alternative valve area calculation formula:**

**AVAproj = AVArest + (AVApeak - AVArest / Qpeak - Qrest) × (250 - Qrest)**

where AVArest is the aortic valve area measured by the continuity equation at rest in cm^2^, AVApeak is the aortic valve area measured by the continuity equation at peak dobutamine infusion in cm^2^, Q_rest_ is the alternative measurement of flow at rest expressed in mL/s, and Q_peak_ is the alternative measurement of flow at peak dobutamine infusion expressed in mL/s.

## Figures and Tables

**Figure 1:**
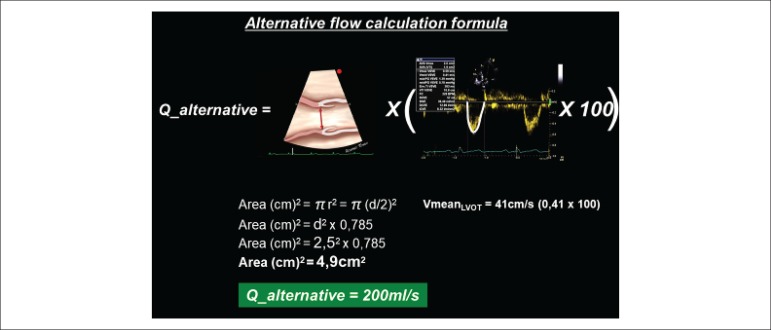
Alternative flow calculation formula where: Q_alternative_ is flow in mL/s, AST_LVOT_ is the sectional transverse area of the left ventricular outflow tract (LVOT) in cm^2^, and Vmean_LVOT_ is the mean blood flow velocity by pulsed wave Doppler at the LVOT level during left ventricular ejection, being expressed in m/s.

**Figure 2:**
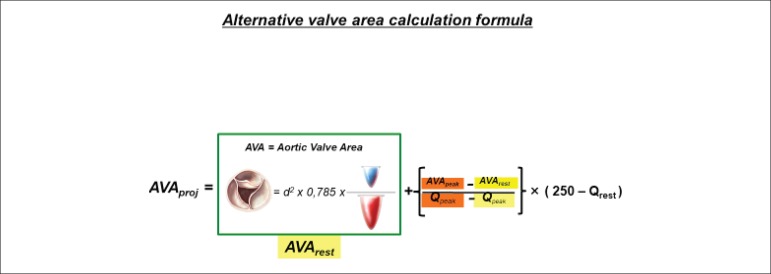
Alternative valve area calculation formula where: AVArest is the aortic valve area measured by the continuity equation at rest in cm^2^, AVApeak is the aortic valve area measured by the continuity equation at peak dobutamine infusion in cm^2^, Q_rest_ is the alternative measurement of flow at rest expressed in mL/s, and Q_peak_ is the alternative measurement of flow at peak dobutamine infusion expressed in mL/s.
